# Spatial Distribution of Precipitation in Huang-Huai-Hai River Basin between 1961 to 2016, China

**DOI:** 10.3390/ijerph16183404

**Published:** 2019-09-13

**Authors:** Yong Yuan, Denghua Yan, Zhe Yuan, Jun Yin, Zhongnan Zhao

**Affiliations:** 1 General Institute of Water Resources and Hydropower Planning and Design, Ministry of Water Resources, Beijing 100120, China; yuany@giwp.org.cn (Y.Y.); zhaozhongnan@giwp.org.cn (Z.Z.); 2 Department of Water Resources, China Institute of Water Resources and Hydropower Research, Beijing 100038, China; 3 Changjiang River Scientific Research Institute, Changjiang Water Resources Commission of the Ministry of Water Resources of China, Wuhan 430010, China; yuanzhe_0116@126.com; 4 Faculty of Resources and Environmental Science, Hubei University, Wuhan 430062, China; yinjun19880209@126.com

**Keywords:** Huang-huai-hai River Basin, precipitation classes, precipitation cover area, extreme precipitation, IDW (Inverse Distance Weighting), generalized extreme-value distribution

## Abstract

The Huang-huai-hai River Basin is one of the most economically developed areas, but is also heavily impacted by drought and flood disasters. Research on the precipitation feature of the Huang-huai-hai River Basin is of great importance to the further discussion of the cause of flood disaster. Based on the selected meteorological stations of the study area from 1961–2016, the inverse distance weighting method was used to get daily precipitation grid data. Interannual variation of precipitation intensity and cover area of different precipitation classes was analyzed. The generalized extreme-value distribution method was used to analyze the spatial distribution of extreme precipitation. The results show that: (1) decrease of accumulated precipitation in light precipitation year and moderate precipitation year might be the reason why the precipitation in the whole basin decreased, but the coefficient of variation (CV) of different classes of precipitation and precipitation days does not change significantly; (2) since the cover area of precipitation > 50 mm and precipitation intensity both decreased, the extreme precipitation of the whole basin may be decreasing; (3) extreme precipitation mainly occurred in the loess plateau in the northeast of Huang-huai-hai River Basin, Dabieshan in the middle of Huang-huai-hai River Basin and other areas.

## 1. Introduction

The Huang-huai-hai River Basin, which includes three larger rivers, the Yellow River, Huaihe River and Haihe River, is one of the most economically developed areas, but is also heavily impacted by flood disasters which have brought about great economic losses [1,2]. In 2013, economic losses in the Huang-huai-hai River Basin caused by flood was about 60 billion RMB [3]. Flood disaster has posed severe threat to the security of local residents. Precipitation is the main causing factor of flood. Many scholars have done research on the relationship between precipitation feature and flood [4,5,6,7]. Pielke presented a conceptual framework which assessed the role that variability in precipitation had in damaging flooding in the United States at national and regional levels [8]; Hartmann suggested that strong monsoon precipitation in the arid high mountainous regions played a major role in the 2010 floods in Pakistan [9]. Drought seriously restricts the economic and social development of the Huang-huai-hai River Basin due to water resource shortage, and some researchers have studied the drought feature and its impacts [10,11,12]. Yuan et al. shows that the Huang-huai-hai River Basin mainly experienced moderate drought and severe drought, and consecutive drought of several seasons often took place in the Ningxia plain [13]. However, all the research above is from temporal scale.

As research and climate change continue, there is growing recognition that extreme precipitation (frequency, intensity and time) has great impact on flood disaster [14,15,16]. Norbiato et al. analyzed the relationship between extreme precipitation and flash flood on 29 August 2003 in the upper Tagliamento River Basin, Eastern Italian Alps [17]. Kwon et al. utilized a weather-state-based, stochastic multivariate model as a conditional probability model to simulate the natural precipitation field, so as to calculate design flood, and suggested that large-scale climatic patterns serve as a major driver of persistent year-to-year changes in precipitation probabilities [18]. A lot of methods analyzing extreme precipitation have also been brought out. For example, the Expert Team on Climate Change Detection and Indices (ETCCDI) suggested 27 core extreme indices based on daily temperature and precipitation, including maximum one-day precipitation, maximum five-day precipitation, tropical nights, and so on [19,20]. Xia et al. suggested that the precipitation in the upper and middle reaches of the Yellow River reduced obviously in spring and summer, but increased significantly in autumn, especially after 2000 [21]. For extreme precipitation in Huaihe River Basin, research shows that extreme precipitation did not have significant changing trend [22,23]. In the Haihe River Basin, Li et al. argued that light rain was the dominant precipitation, and the change of precipitation intensity was not significant [24]. However, the above-mentioned research usually analyzes extreme precipitation of meteorological stations and then draws extreme precipitation feature maps. There is a shortage of research on spatial distribution of extreme precipitation.

Based on the precipitation data of the Huang-huai-hai River Basin, this research analyzed the variation character of intensity and cover area of different classes of precipitation in the study area. Besides this, spatial distribution of extreme precipitation was also analyzed. This research is expected to provide reference for the discussion of the reason for flood disaster in the Huang-huai-hai River Basin from two angles: Variation character of precipitation event and spatial non-uniformity of extreme precipitation.

## 2. Materials and Methods

The total area of Huang-huai-hai River Basin is about 1.433 × 10^6^ km^2^ with east-west length of 2344.5 km and north-south width of 1323.6 km. The geographic scope of the study area is 95°53′~122°60′ E, 32°10′~43° N. The elevation of the Huang-huai-hai River Basin decreases from the west to the east in the form of three steps. The first step is the Tibet Plateau with an average elevation of 4000 m; the second step mainly consists of plateau with an average elevation of 1000–2000 m; the third step mainly consists of low mountains, hills and plains with an elevation below 500 m, while the elevation of the North China Plain is less than 200 m. Precipitation of the Huang-huai-hai River Basin is mainly impacted by the intensity of pacific monsoon and the forward and withdraw of rain belt, thus has an uneven spatial distribution and violent seasonal and interannual change. Annual average precipitation of the whole region is 471 mm to 864 mm. The population of the Huang-huai-hai River Basin is about 35% of China, and its GDP is about 32% that of China [25]. The Huang-huai-hai River Basin is an important agriculture production base of China, whose cultivated area and grain yield are about 20.4% and 23.6% of China, respectively [26].

### 2.1. Meteorological Data Source

**Figure 1:**
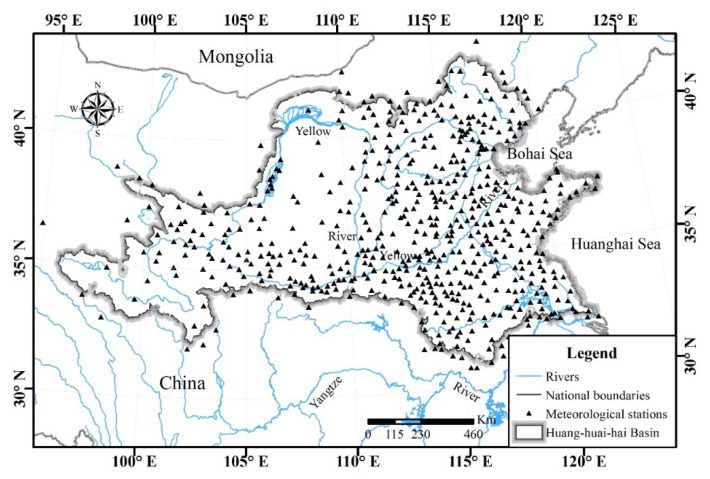
Location of the Huang-huai-hai River Basin and the meteorological stations.

The meteorological data used in this research comes from China Meteorological Data Sharing Service System. Daily precipitation (1 January 1961–31 December 2016) with good consistency (there are less than 10 days from which data are absent) from 566 meteorological stations within and around the Huang-huai-hai River Basin was used in this research (Figure 1). Based on the above-mentioned data, inverse distance weighting (IDW) [27] was used to do spatial interpolation so as to obtain daily precipitation grid data with the resolution of 5 km × 5 km. The value of the estimated point is obtained by weighted averaging of the distance between the estimated point and the sample point. The closer the sample point to be evaluated is, the greater the weight it is given.

### 2.2. Method

To do summation of daily precipitation grid data in order to get yearly precipitation grid data. Yearly precipitation and rainy days of Huang-huai-hai River Basin during 1961–2016 were calculated. Based on annual precipitation and rainy days, CV was calculated according to the following equation [28,29].

(1) $$\mathrm{CV}=\frac{\sigma }{\mu }\times 100$$

where, CV is coefficient of variation; σ is standard deviation; μ is average vale.

Besides this, the slope of liner regression equation of precipitation and rainy days of each grid were calculated according to the following equation [30].

(2) $$Slope=\frac{n\times \sum_{i=1}^{n}\left(i\times K_{i}\right)-\sum_{i=1}^{n}i\sum_{i=1}^{n}K_{i}}{n\times \sum_{i=1}^{n}i^{2}-{\left(\sum_{i=1}^{n}i\right)}^{2}}$$

If *Slope* > 0, it means the precipitation/rainy days were increasing during the study period; while *Slope* < 0 means they were decreasing. Slope is the link-line between the first year and the last year, it also reflects the general changing trend of the average precipitation/rainy days in each grid calculated by liner regression equation during the study period. *n* stands for total years, and *K_i_* is the precipitation/rainy days in *i^th^* year.

According to the precipitation intensity classification standard raised by China’s Meteorological Administration [31], precipitation of the Huang-huai-hai River Basin is classified into 5 grades: light rain, mild rain, heavy rain, very heavy rain and extreme precipitation (Table 1). Based on the daily gridded precipitation data, yearly precipitation, rainy days, precipitation intensity (divide annual precipitation by rainy days), ratio of precipitation, multi-year average precipitation, ratio of rainy days and the whole number of days in a year (365 days or 366 days) and cover area of precipitation of each class during the study period were all calculated.

Generalized Extreme-Value (GEV) is widely used in the research on extreme precipitation variation [32,33]. Total extreme precipitation would obtain exceeding special threshold (95th and 99th percentile was threshold in this article) by GEV to study spatial pattern of extreme precipitation. The 95th percentile and 99th percentile of the precipitation in grid data were calculated by GEV distribution to get a corresponding threshold map. Based on the threshold map, total precipitation which exceeds the threshold during the study period was calculated; by dividing this amount of precipitation by multi-year average precipitation, we can get the standardized extreme precipitation distribution map.

## 3. Results

### 3.1. Interannual Variation of Precipitation

**Figure 2:**
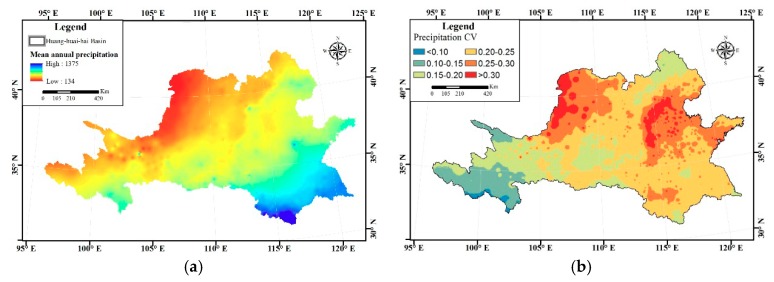
(**a**) Multi-year average precipitation; (**b**) Coefficient variance of precipitation in Huang-huai-hai River Basin.

**Figure 3:**
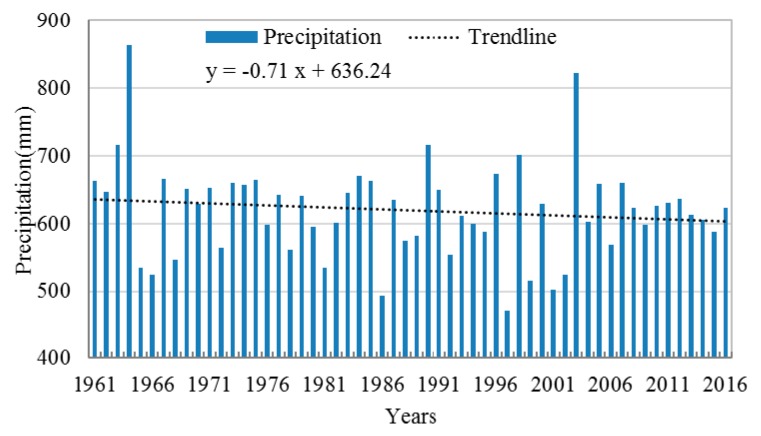
Interannual variation of precipitation in Huang-huai-hai River Basin.

Multi-year average precipitation of the Huang-huai-hai River Basin is shown in Figure 2a. In the grid, the maximum precipitation occurs in the south of the Huang-huai-hai River Basin with 1375 mm, while the minimum precipitation occurs in the north of the Huang-huai-hai River Basin with 134 mm. The maximum precipitation is about 10 times the minimum precipitation. It can be seen here that the precipitation of Huang-huai-hai River Basin shows a decreasing trend on the whole, which is about −0.71 mm/annually, as seen in Figure 3.

By calculating CV (coefficient of variation) of precipitation spatial change in Huang-huai-hai River Basin (Figure 2b), it can be determined that the maximum CV is 0.39 and the minimum CV is 0.09. The region where CV is larger than 0.25 is mainly located in the Hetao Plain of the north, North China Plain of the east and upper reaches of the Huaihe River of the south. Table 2 shows that the minimum CV of decadal precipitation occurs in mild rain with about 0.3, while the maximum CV happens to very heavy rain with about 2.3. The minimum CV of decadal rainy days occurs in light rain with about 0.3, while the maximum CV also occurs in heavy rain with about 2.3 (Table 3).

**Figure 4:**
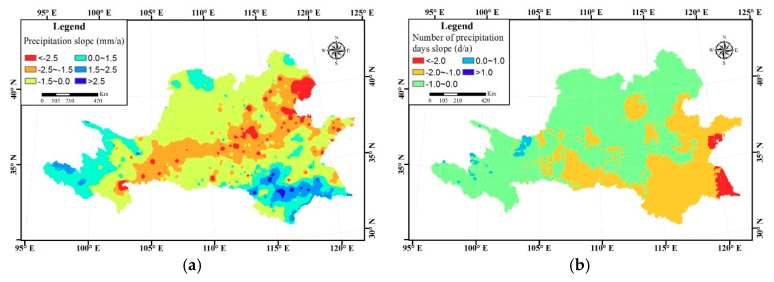
(**a**) Slope of precipitation; (**b**) rainy days.

Figure 4 shows the slope of precipitation and rainy days. Figure 4a shows that there is a precipitation belt in the middle of the Huang-huai-hai River Basin which has an obvious decreasing trend. On the other hand, precipitation of the Huaihe River Basin in the south of the study area has an obvious increasing trend. For rainy days, decreasing trends mainly take place in the east and south part (Figure 4b). Precipitation in the Huaihe River Basin of the south of the study area increases while the rainy days decrease. Thus, it can be speculated that the precipitation intensity of this area may increase, and the probability of extreme precipitation occurrence may also increase. Influenced by monsoon, the Huaihe River Basin has always been one of the regions which is heavily affected by flood. The precipitation intensity and frequency of extreme precipitation are also comparatively large [34,35].

### 3.2. Interannual Variation of Precipitation Intensity for Each Grade

**Figure 5:**
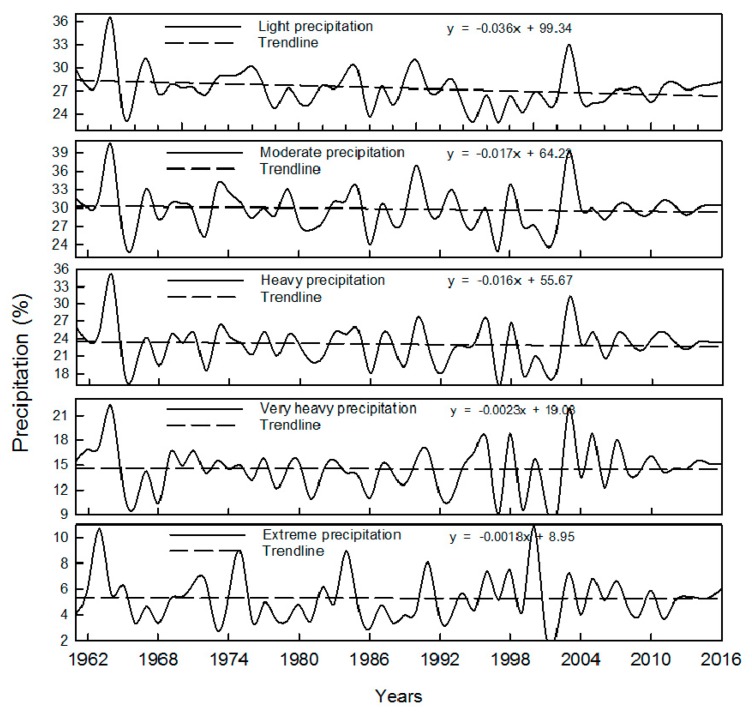
Ratio between precipitation of each grade and total precipitation of a whole year.

**Figure 6:**
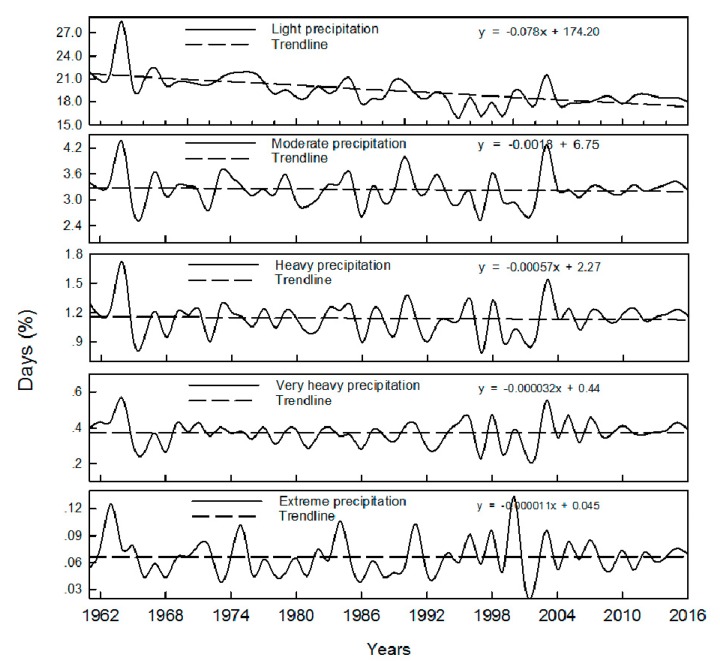
Ratio between rainy days of each grade and the total number of days in a year.

Ratio between precipitation of each grade and total precipitation of a whole year is shown in Figure 5. It can be seen that multiannual average ratio (the same below) between light rain and total precipitation of a whole year is about 27%, with a decreasing trend; the ratio between mild rain and total precipitation of a whole year is about 30%, also with an decreasing trend; the ratio between heavy rain and total precipitation of a whole year is about 23%, with a slightly decreasing trend; the ratio between very heavy rain and total precipitation of a whole year is about 14%, with no significant changing trend; the ratio between extreme rain and total precipitation of a whole year is about 5%, also with no significant changing trend. Figure 6 shows the ratio between rainy days and the total number of days in a year (365 days or 366 days). It can be seen from Figure 6 that rainy days for light rain is about 19% of the total number of days in a year, with an decreasing trend; rainy days for mild rain is about 3% of the total number of days in a year, with a decreasing trend; rainy days for heavy rain is about 1% of the total number of days in a year, with a slightly decreasing trend; rainy days for very heavy rain is about 0.3% of the total number of days in a year, with no significant changing trend; rainy days for extreme rain is about 0.06% of the total number of days in a year, also with a decreasing trend. Considering the situation that the precipitation has been decreasing each year in the study area, as shown in Figure 3, the decrease of precipitation and rainy days for light rain and mild rain may be the cause of the decrease of whole precipitation in the study area.

**Figure 7:**
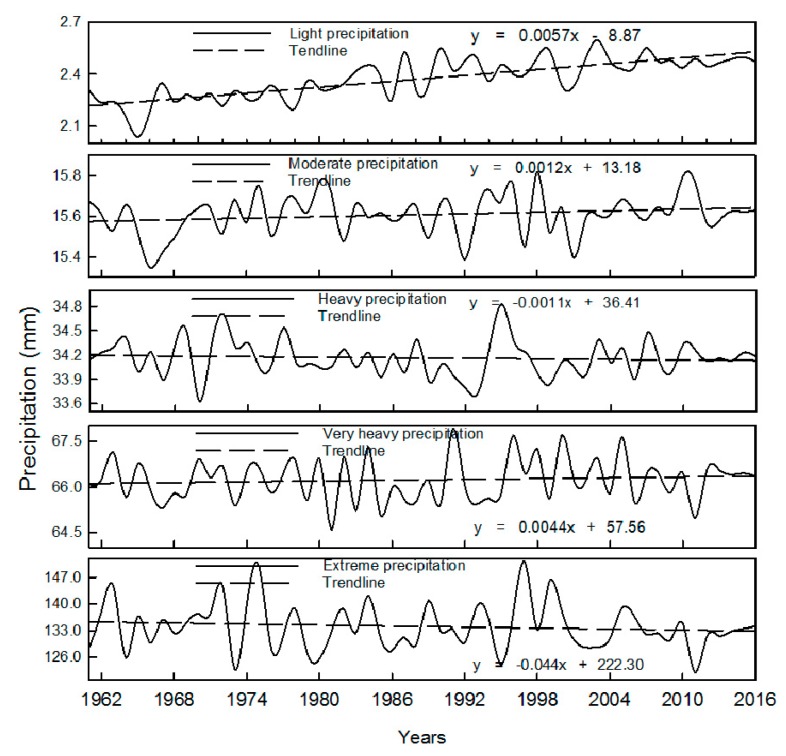
Interannual variation of precipitation intensity of each grade.

Figure 7 shows the interannual variation of precipitation intensity of each grade. It can be seen that the intensity of light rain and mild rain has an increasing trend; the intensity of heavy rain and very heavy rain has no significant changing trend; the intensity of extreme rain has a decreasing trend. The results have also been presented by others [36,37].

### 3.3. Interannual Variation of Precipitation Cover Area for Each Grade

**Figure 8:**
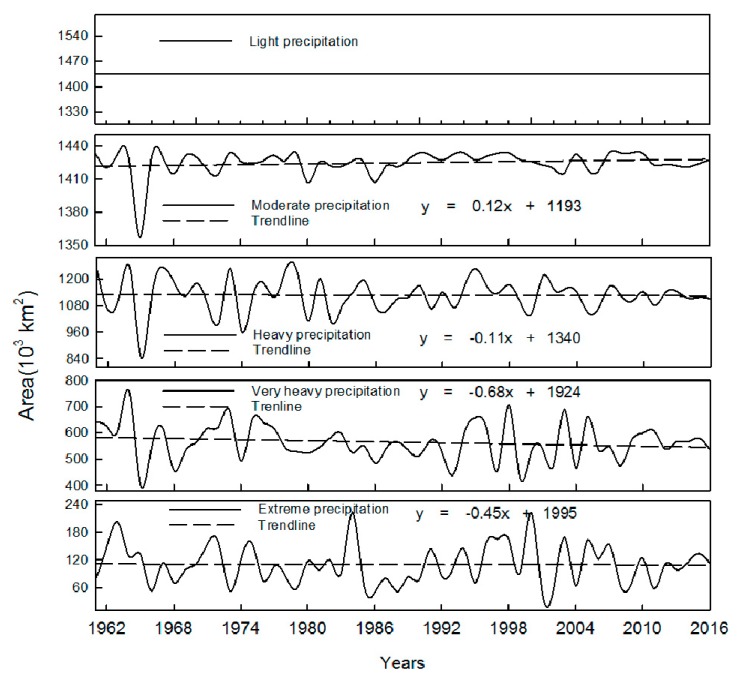
Interannual variation of precipitation cover area for each grade.

Interannual variation of precipitation cover area for each grade is shown in Figure 8 and Table 4. From Figure 8 it can be seen that light rain covers the whole study area, with an area of about 1440 × 10^3^ km^3^; mild rain covers an area of about 1425 × 10^3^ km^3^, with an increasing trend; the heavy rain, very heavy rain and extreme rain cover an area of about 1127 × 10^3^ km^3^, 565 × 10^3^ km^3^ and 110 × 10^3^ km^3^ respectively, all with a decreasing trend. Table 4 shows that the precipitation cover area of each grade basically all reached the top in the 1990s. Considering that the intensity of extreme rain has a decreasing trend (Figure 7), the extreme precipitation in the whole study area may decrease on the whole, which is similar to the research done by Zhang et al. [38].

### 3.4. Spatial Distribution of Extreme Precipitation

**Figure 9:**
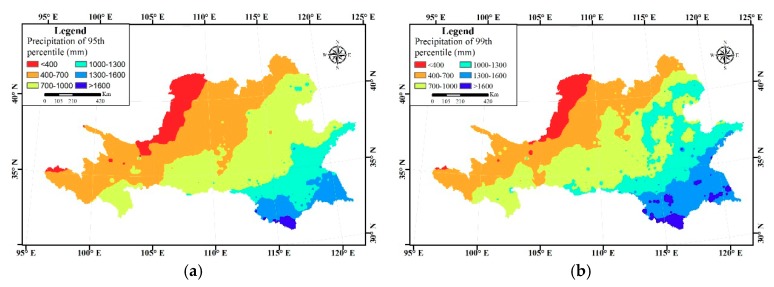
The 95th percentile and 99th percentile of the extreme precipitation threshold. (**a**) The 95th percentile; (**b**) the 99th percentile.

**Figure 10:**
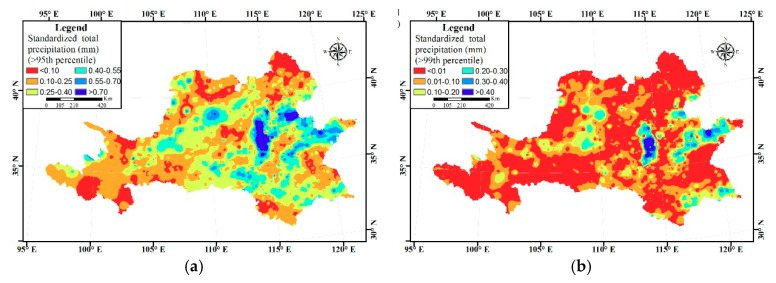
The standardized extreme precipitation accumulation larger than the 95th percentile and 99th percentile. (**a**) The 95th percentile; (**b**) the 99th percentile.

The 95th percentile and 99th percentile of the extreme precipitation threshold are shown in Figure 9. It can be seen that the threshold in both pictures is in the shape of about five belts, increasing from the northeast to the southwest. If we assume that the area where the standardized extreme precipitation accumulation, as seen in Figure 10, is larger than 0.4 (i.e., the blue part in Figure 10) is the region suffering from extreme precipitation, the extreme precipitation is most severe in the Loess Plateau of the northeast, Dabieshan area of the mid Shandong peninsula of the east, middle and lower reaches of the Huaihe River Basin of the south.

## 4. Conclusions

Based on the data of meteorological stations in the Huang-huai-hai River Basin, the amount of precipitation and the temporal-spatial distribution of each precipitation grade (light rain, mild rain, heavy rain, very heavy rain and extreme rain) of the study area during 1961–2016 were analyzed via daily interpolated meteorological data. Main conclusions are as follows:

(1) The precipitation of the Huang-huai-hai River Basin showed a decreasing trend; the amount of precipitation of each grade and CV for rainy days did not change much; Huai-he River Basin in the south of the study area is at high risk of extreme precipitation. Considering serious shortage of water resources and a high degree of water resources development and utilization, the reduction of precipitation would aggravate effects of drought in this basin.

(2) The ratio between the light/mild precipitation amount and the total precipitation amount, and the ratio between the light rain/mild rainy days and the total number of days are comparatively big, but they both have decreasing trends, which could be the main reason for the decrease of total precipitation amount. Because of the special underlying surface in the basin, short-duration, light and moderate precipitation usually have no runoff observed.

(3) The cover area of the heavy rain, very heavy rain and extreme rain all showed a decreasing trend. Considering the decreasing trend of the extreme rain intensity, the extreme precipitation amount in the whole study area may also decrease.

(4) In the Huang-huai-hai River Basin, extreme precipitation, which analyzed by generalized extreme-value (GEV), was most severe in Loess Plateau of the northeast, Dabieshan area of the mid-Shandong peninsula of the east, middle and lower reaches of the Huaihe River Basin of the south. These areas are high-risk regions of flood disaster.

The above-mentioned conclusions are of great importance to reveal the variation of precipitation, cause of drought/flood and coping measures. Since the area of the Huang-huai-hai River Basin is big and the duration and cover area of extreme precipitation is small, the precision for the extreme precipitation of this research might need further improvement.

## Figures and Tables

**Table 1:** Grade of precipitation [31] (unit: mm).

	Light Precipitation	Moderate Precipitation	Heavy Precipitation	Very Heavy Precipitation	Extreme Precipitation
Precipitation	P24 < 10	10 ≤ P24 < 25	25 ≤ P24 < 50	50 ≤ P24 < 100	100 ≤ P24

Note: P24 means total precipitation of 24 h.

**Table 2:** Interannual variation of CV of precipitation.

	1961–1969	1970–1979	1980–1989	1990–1999	2000–2009	2010–2016
Light	0.44	0.45	0.46	0.45	0.45	0.45
Moderate	0.33	0.32	0.32	0.32	0.33	0.32
Heavy	0.53	0.53	0.56	0.53	0.53	0.54
Very heavy	1.00	0.97	1.03	1.03	1.02	1.01
Extreme	2.29	2.33	2.42	2.22	2.35	2.32

**Table 3:** Interannual variation of CV of rainy days.

	1961–1969	1970–1979	1980–1989	1990–1999	2000–2009	2010–2016
Light	0.29	0.29	0.30	0.30	0.28	0.29
Moderate	0.41	0.41	0.45	0.41	0.43	0.42
Heavy	0.66	0.68	0.71	0.69	0.67	0.68
Very Heavy	1.12	1.09	1.16	1.16	1.14	1.14
Extreme	2.33	2.33	2.48	2.25	2.40	2.36

**Table 4:** Interannual variation of precipitation cover area for each grade.

	1961–1969	1970–1979	1980–1989	1990–1999	2000–2009	2010–2016
Light	1434.03	1434.03	1434.03	1434.03	1434.03	1434.03
Moderate	1419.63	1426.77	1420.55	1431.26	1426.07	1425.01
Heavy	1135.75	1138.30	1099.90	1143.21	1121.08	1127.23
Very Heavy	576.56	596.13	544.98	552.14	558.15	565.08
Extreme	113.71	107.63	98.41	120.46	109.39	109.83
